# Port Workers’ Use of Medical Services in a Maritime Container Terminal in Costa Rica

**DOI:** 10.3390/ijerph20021124

**Published:** 2023-01-08

**Authors:** Alejandro Martínez, Olaf C. Jensen

**Affiliations:** 1 Department of Maritime Health, College of Physicians and Surgeons, Sabana Sur, San José CP 10109, Costa Rica; 2 Centre for Maritime Health and Society, Department of Public Health, University of Southern Denmark, Degnevej 14, 6705 Esbjerg, Denmark

**Keywords:** medical attention, alcohol and drug control, doping, workplace prevention, occupational epidemiology, cost–benefit analysis

## Abstract

Supervised by three or four medical doctors and one nurse in rotating shifts, the medical clinic in Costa Rica’s Moín Container Terminal is open 24/7 for visits from port workers. In our study, we aimed to identify the sociodemographic and clinical characteristics of a consecutive series of patients who attended the medical clinic for outpatient services during an 8-month period. Our descriptive study involved collecting patient records from the medical clinic during the first 8 months of 2021 (i.e., 1 January–31 August 2021), during which 3050 visits from 1301 port workers were registered. Terminal tractor drivers, crane operators, and stevedores were the most frequent job categories among the patients. Doping (i.e., ICD-10 Z03.6) was observed in 64% of the visits. The top ICD-10 codes among all other patients not observed to have engaged in doping (n = 469) were diseases of the musculoskeletal system (7.2%) and abnormal clinical and laboratory symptoms (6.2%). Problems with the musculoskeletal system were primarily back pain (36.0%), muscle contracture (30.1%), and secondary headache (25.2%). Two-thirds of the visits were due to screening for alcohol and drugs or doping; however, inconsistency in the coding system complicates the analysis of data, and a dropdown menu in the registration is therefore needed to prevent errors. Relative risk calculations are impossible due to a lack of data about the at-risk population but should be pursued under different circumstances in future studies. In the support chain of goods, the medical clinic in the port plays a key role in saving time in shipping, which means that the injured or sick employees in most cases can continue working. For the shipping industry, quick un- and offloading is very important to stay competitive in the market for transport.

## 1. Introduction

Today, little more than 50,000 merchant ships carry 90% of all trade. The people responsible for maintaining, running, and operating the fleet are seafarers, who together ensure that essential household items—televisions, laptops, and clothing, to name a few—are brought by sea to ports for consumers [1]. Port workers play a pivotal role in the supply chain by loading and unloading the same 90% of global trade, as well as by preparing docks for incoming ships, mooring ships correctly upon arrival or departure, maintaining accurate records of damaged goods in a timely manner, and performing the field operation of storehouses, transport in the port, and other processes of production. Port workers perform those central tasks with cranes and terminal tractors near the front of the quay as well as in the warehouse, yard, and other zones, while stevedores mainly operate on board ships and stow cargo [2,3].

The APM Terminal is in Limón, a city in eastern Costa Rica on the Caribbean coast close to the MCT with a population of approximately 98,848 as of 2017. Limón is one of the country’s most important port cities and has played host to the MCT since February 2019. 

APM Terminals operates one of the world’s most comprehensive port networks at Costa Rica’s Moín Container Terminal (MCT), which is built on an artificial island off Costa Rica’s Caribbean coast [4].

Operational since February 2019, the MCT is one of the most efficient ports in Latin America and has generated nearly 1000 new jobs, 95% of which have been filled by workers from the province of Limón. As such, the MCT has provided outstanding stability for APM Terminals, since the majority of approximately 1000 have continued working with them as they have done from the start in 2019.

Since port workers are usually on their feet and manage heavy cargo cranes and tractors around the clock, day in and day out, they must be in excellent physical condition [5,6]. For that reason, it is a public health concern to maintain a medical clinic located within the terminal area that is open for service 24/7, and that looks for improvements of the occupational environment.

MCT port workers have access to at least four different health systems: the national social security for nonoccupational diseases, a public insurance system for work-related diseases or accidents, a private medical insurance that is provided by APM for any kind of medical problem, and a medical clinic within the terminal area. It is important to point out that some medical issues must be reported to the social security or to the public insurance system, according to the laws of the country. Therefore, certain medical cases cannot be handled at all or only partially by the AMP medical clinic. 

The clinic within the terminal provides free medical services: emergency care attendance, control of chronic diseases, prenatal care control, drug and alcohol screening, and so on; however, APM management may determine which services can be provided.

To offer around-the-clock coverage, the MCT’s rotating shift pattern entails three 8 h shifts, usually (I) 06:00–14:00, (II) 14:00–22:00, and (III) 22:00–06:00. The clinic receives patients 24/7 and records approximately 4500 visits every 12 months. 

The staff of the MCT’s medical clinic consists of four doctors, who split three shifts from Monday to Sunday, and a part-time nurse, who works from Monday to Friday. A Red Cross driver and a paramedic are also present during each shift to assist with transfers and emergencies.

The employees are randomly selected for drug and alcohol screening, usually by a security staff member or according to management strategies. A breathalyser and a rapid drug testing kit are the methods used to perform the procedure. If a test result is positive, the employee is referred to a national institution that provides medical care for persons who suffer addictive disorders and/or alcoholism.

The medical clinic provides the advantage of having a rapid response team and professionals who can provide occupational feedback and/or implement measures that produce an effect upon identified risk factors.

To our knowledge, a type of study like the one we present here has never been undertaken. In response, to help to improve the quality of other clinics, in our study, we sought to illuminate important characteristics of the port workers’ use of the medical clinic at the MCT. We aimed to identify the sociodemographic and clinical characteristics of a consecutive series of patients who attended the clinic for outpatient services in an 8-month period. To identify patterns of the occurrence of disease based on patient records regarding all visits to the MCT’s medical clinic during the first 8 months of 2021, we conducted a descriptive study. In this article, we discuss the use of such data in relation to prevention and the development of a systematic clinical database.

## 2. Materials and Methods 

We used the records of the medical consultations of the port workers. We also interviewed the doctors of the clinic to clarify the process that they used to file the information that we used for our study.

We included headache as a musculoskeletal disorder because, according to MCT doctors, that code was used for secondary headaches due to cervicalgia or dorsalgia. If a primary headache was diagnosed, the report appears as migraine. 

When more than two drugs of different families were indicated, we classified the case according to the most representative drug based on the pathology. For example, if the consultation was for low back pain and dexamethasone and dexketoprofen were prescribed, the patient was recorded as having received NSAIDs. 

We decided to report the most representative medication group in consonance with the medical diagnosis when two or more drugs were prescribed in the consultations; for instance, if histamine H2 receptor antagonists and proton-pump inhibitors appeared on file, we reported them as antacids. Besides, hydration includes oral rehydration salts and saline solution. 

### 2.1. Inclusion Criteria

All port workers (n = 3050) who sought medical attention in the clinic from 1 January to 31 August were included, however, some patients attended to the clinic multiple times. Therefore, for our frequency analysis of the variable, patients that attended the clinic two times or more were excluded of the analysis.

The records of the patients that we used cover all the individuals that attended the medical clinic searching for help for any medical issue or any administrative examination.

### 2.2. Measures

The nurse or attending doctor collected patients’ information by interviewing all patients immediately upon their arrival at the clinic, and all information was coded in accordance with the hospital emergency room data registration system. 

Fourteen variables were recorded in the clinic for each patient in a predesigned Excel sheet: date, week, gender, age, hour of the day, medical doctor attending, rotating shift (i.e., I, II, or III), work department, position, ICD-10 diagnostic group, name of disease, medical problem presented, medical treatment administered, and follow-up plan. Fatal injuries were not included, and all personal data in the data set used were deleted from our analysis. For drug and alcohol testing, we used the ICD-10 code Z03.6 (i.e., observation of a suspected toxic effect from an ingested substance). 

### 2.3. Statistical Analysis

All data were analyzed using SPSS version 28.0 (IBM, Armonk, NY, USA), encoded into categorical data, and presented in tables within this article. Descriptive analysis were used to identify basic characteristics of the data and to capture the percentage distribution of responses related to demographic background, work area, and job duties. 

### 2.4. Protection of Personal Data

We handled data in accordance with Costa Rican privacy laws, which follow the European Union’s General Data Protection Regulation (GDPR) model. Costa Rica is currently engaged in a comprehensive reform of its data privacy laws based on the GDPR model. All potentially identifying information in the data was removed so that the data could be aggregated to a level at which all patients were anonymous and cannot be identified as individual patients. Such statistical information is not covered by the GDPR [7].

## 3. Results

The total sample comprised 3050 visits to the clinic from 1 January to 31 August 2021 made by 1301 different patients. By gender, 12.1% of patients were women and 87.9% were men; by age, 22% were 20- to 29-year-olds, 62% were 30- to 49-year-olds, and 16% were at least 50 years old. As for nationality, most patients were from Costa Rica (97.7%), whereas others were from Nicaragua (1.0%) or other neighboring countries. Of all 1301 patients, 832 (64.0%) visited the clinic for random screening for drugs and alcohol (i.e., doping) according to ICD-10 Z03.6 (i.e., observation of a suspected toxic effect from an ingested substance). The top job categories among the patients were terminal tractor driver (18.3%), crane operator (18.1%), stevedore (11.3%), safety manager (6.0%), and reefer assistant or reefer technician (6.2%). The majority of patients consulted during day hours. Most of the workers were men.

### 3.1. Subanalysis of the ICD-10 Groups

The top ICD-10 codes among the 469 patients who did not visit the clinic for alcohol and drug screening were diseases of the musculoskeletal system (19.8%), factors influencing the health status and contact with health services (17.5%), and abnormal clinical and laboratory symptoms (17.0%), detailed in Table 1. Problems with the musculoskeletal system were primarily back pain (36.1%), muscle contracture (30.2%), and headache (25.3%).

### 3.2. Subanalysis of Patients Who Needed Medical Treatment

The types of medicaments prescribed to 326 of the 469 patients who needed medical treatment were nonsteroidal anti-inflammatory drugs (NSAIDs) (51.5%), antispasmodics (9.5%), antihistamines (6.4%), glucocorticoids (6.1%), and antacids (5.5%) (Table 2). 

### 3.3. Subanalysis of the Largest ICD-10 Group, Test for Doping ICD-10 Z03

The top job categories with the highest proportions in the sample—recorded for test for doping, ICD-10 Z03.6 (i.e., observation of a suspected toxic effect from an ingested substance)—were terminal tractor drivers (n = 164/239, 68.6%), crane operators (n = 189/235, 80.3%), and stevedores (n = 116/147, 78.9%) (Table 3). 

### 3.4. Subanalysis of the Percentage of Consultations per Shift

There were a bigger number of medical consultations (38%) during the first shift, 06:00–14:00. (Table 4)

### 3.5. Subanalysis of the Ages of Workers

Most workers were between 30 and 49 years old (Table 5).

### 3.6. Gender of the Employees

About 88% of the workers were men (Table 6).

## 4. Discussion

We report clinical data from a medical clinic located inside a large maritime container terminal in Costa Rica, which, to our knowledge, is the first report of its type. Among the key findings were 62% of the patients were 30–49 years old, 97.7% were from Costa Rica, and the most frequent reason for the visits was alcohol and drug screening (64%), whereas all other reasons were in a wide spectrum of conditions in ICD-10. The top 3 ICD-10 diagnoses among the 468 patients who did not visit the clinic for alcohol and drug screening were diseases of the musculoskeletal system (19.8%), factors influencing the health status and contact with health services (17.5%), and abnormal clinical and laboratory symptoms (17.0%). 

Problems with the musculoskeletal system were primarily back pain (36.1%), muscle contracture (30.2%), and headache (25.3%). The findings have important implications for understanding patterns of medical claims among port workers and, in turn, improving the quality and effectiveness of medical services at such clinics and workplace prevention.

### 4.1. Direct Relationship between the Profession of the Port Worker and Low Back Pain

Musculoskeletal disorders occur frequently among port workers. According to the National Insurance Institute of Costa Rica, from 2007 to 2008, low back pain was the most frequent cause of disability among port workers aged more than 45 years old [8]. For workers, using NSAIDs (e.g., diclofenac) to treat such pain decreases the numbers of days on which they are unable to work. In one study, workers without work-related musculoskeletal disorders had significantly higher scores for quality of life than for functional capacity, physical and social aspects, pain, and vitality [9]. 

On that topic, obtaining adequate pre-employment evaluation is essential for workplace prevention. Another preventive strategy is to perform routine risk assessments of the workplace and replace nonergonomic machinery, including the seats of the terminal tractors. Osteoarthrosis and other causes of musculoskeletal pain can be superimposed on occupational diseases, thereby making it difficult to diagnose occupational disease or injury secondary to a workplace accident. In Costa Rica, occupational assessments are usually brief and lack scientific rigidity. Medical doctors should be trained in occupational medicine, have knowledge of workplace risks, and report any occupational disease to the insurance company [9].

### 4.2. Observation of Suspected Toxic Effects of Ingesting Substances (i.e., Doping)

Doping, or ICD-10 code Z03.6 (i.e., Observation of a suspected toxic effect from an ingested substance), was by far the most common condition in the sample (*n* = 832/1301 patients, 64%). A subanalysis of the most populous job categories—that is, crane operators (n = 235) and stevedores (n = 147)—showed that 80.4% and 78.9% of their visits to the clinic, respectively, were for drug and alcohol screening. Beyond that, in another study, of 232 port workers in Rio Grande do Sul, Brazil, 29 reported using illegal drugs [10]. Those findings are relevant because such workers handle powerful machines with a high risk of causing injury to people working under or close to them. In response, randomly testing workers for doping has been shown to reduce the risk of workplace accidents [11]. 

Even so, the maritime transportation of illegal drugs from South America is another serious problem. Various publicly available sources suggest that the estimated number of drug shipments initiated per month ranges from 4 to 72, and at any given time, two to four vessels of all types on the high seas are carrying illegal drugs. In response, the United States continues to invest considerable effort in searching and interdicting drug trafficking vessels in the Caribbean and Eastern Pacific regions [12]. Regarding drug screening, the average value of each test and what it represents in terms of economic burden for the clinic, as well as the percentage of labor hours that they represent, should be studied to support the use of such tests or guide how they should be used. 

### 4.3. Comments on the Use of Medical Treatments 

The distribution of the types of medical treatment (n = 326) of patients who needed medical treatment is in Table 2. 

In social security, muscle relaxants are reserved only for certain specialties, and only primary care physicians are authorized to prescribe anti-inflammatory medication. 

As mentioned, in many consultations, patients received symptomatic treatment; however, it should be considered that the treatment of many musculoskeletal conditions is symptomatic.

The low count of anti-influenza treatments may be due to epidemiological silence because respiratory infections had to be referred to social security to rule out COVID-19.

The low number of antiviral drugs recommended to treat flu may be due to the epidemiological silence provoked by COVID-19. It was mandatory to rule out COVID-19 for most respiratory infections.

Most of the consultations that did not require medication were for administrative procedures or random doping cases or due to accidents. The cases marked “not indicated” refer to inadequate data logging.

The fact that there was only one recorded case of the use of tramadol speaks to the preference and availability of analgesics. When such use is compared with the use in other countries, such as the United States, where opioids are more common, the difference reflects the medical–social circumstances in Costa Rica, where medical doctors are very careful not to induce dependence on medications such as opiates. At the same time, the use of tramadol would imply a positive doping result in random testing, which would subsequently have to be medically justified. In view of the type of consultation given, we found no evidence of other types of medication for chronic diseases, including hypoglycemic agents, neuroleptics, and psychoactive drugs.

### 4.4. Limitations and Directions for Future Research

Our analyses included every patient who attended the clinic during the 8-month observation period, even though most of them had visited two or more times for the same problem. Including only the first visit from each patient allowed for estimating the relative risk for different exposure variables. However, as we do not know the number of workers in the job groups, we cannot estimate the relative risk of, for example, driving dangerous machines under the influence of drugs and alcohol [13]. 

Another limitation was that the accuracy of the data entered was not confirmed, which poses a risk because human error can occur when filling in boxes in patient records. Moreover, albeit with some exceptions, most of the diagnoses were clinical, meaning that there was no assessment of laboratory or clinical tests to support the diagnosis made. 

### 4.5. Cost–Benefit Calculations 

Large workplaces, schools, military, and prisons as examples often have a health clinic inside the institutions to help injured and sick people without leaving the workplace. These clinics are often called infirmaries. In the support chain of goods from ship to port to road transport, the medical clinic in the port plays a key role in saving time in shipping but also in giving good and free treatment to workers. Having an infirmary at the workplace means that the employees do not have to leave the workplace and, in most cases, can continue working. For shipping, quick un- and offloading is very important to stay competitive in the market for transport. 

Future studies should account for the number of workers in specific work tasks to estimate the relative risks in the job groups of, for example, drug testing. Beyond that, interventional studies should be conducted because they allow for assessing the most common complaints and the most frequent pathologies and whether proposed and implemented changes affect the statistics. 

## 5. Conclusions

This study contributes to improving the understanding of medical attention needs among port workers in large maritime container terminals. Two-thirds of the visits to the clinic by port workers were due to screenings for alcohol and drugs; therefore, the use of illegal drugs is an important factor to take into consideration, and better strategies must be undertaken. Our results provide evidence for further research on the topic and guide the treatment of individuals to improve work safety, productivity, and the overall health of the workforce. The calculations of the relative risks for diseases and incidents proved to be impossible due to the lack of data on the number of workers at risk but should be calculated and used in future studies. We observed that inconsistency in the coding system complicates the analysis, and in response, it is recommended to have tested, well-developed dropdown menus in the digitalized registration system to prevent errors. We do not have good data to make a cost–benefit analysis, but it will be possible if there is a better registration and can be recommended. 

## Figures and Tables

**Table 1:** ICD-10 diagnostics distribution among a total of n = 468 patients, exclusive of alcohol and drug screening queries, n = 832.

ICD-10	Main Group	N	%
M00–M99	Diseases of the musculoskeletal system	93	19.8%
Z00–Z99	Factors influencing the health status and contact with health services	82	17.5%
R00–R99	Abnormal clinical and laboratory symptoms	80	17.0%
S00–T98	Injuries, poisoning of external causes	50	10.7%
K00–K93	Diseases of the digestive system	32	6.8%
Z042	Incidents	25	5.3%
J00–J99	Diseases of the respiratory system	18	3.8%
G	Diseases of the nervous system	14	3.0%
H00–H59	Diseases of the eye and its annexes	13	2.8%
A and B	Infectious and parasitic diseases	12	2,5%
H60–H95	Diseases of the ear and the mastoid process	11	2.3%
U072	COVID-19 virus not identified	10	2.1%
N00–N99	Diseases of the genitourinary system	9	1.9%
L00–L99	Diseases of the skin and subcutaneous tissue	8	1.7%
F	Mental and behavioral disorders	7	1.5%
V01–Y98	External causes of morbidity and mortality	6	1.2%
I00–I99	Diseases of the circulatory system	4	0.8%
U12	COVID-19 vaccines causing adverse effects	1	0.2%
Total		468	100%

**Table 2:** Distribution of type of medical treatment (n = 326) of patients who needed medical treatment.

NSAID (1)	168	51.5%
Antispasmodic	31	9.5%
Antihistamine	21	6.4%
Glucocorticoid	20	6.1%
Antacid	18	5.5%
It does not indicate:	17	5.2%
Acetaminophen	15	4.6%
Antibiotic	14	4.3%
Muscle relaxant	9	2.8%
Gastrokinetic	7	2.1%
Antihypertensive	5	1.5%
Weft (material to fill a cavity in a tooth)	1	0.3%
Total	326	100.0%

(1) Non steroid anti-inflammatory drugs.

**Table 3:** ICD-10 distribution of the main patient job groups and main ICD-10 codes (n = 1301).

		Terminal Tractor Drivers (1)		Crane Operators (2)		Stevedores (3)		Total n = 1301	
ICD-10	Risk Factors and Health	N	%	N	%	N	%	N	%
Z03.6	Observation for suspected toxic effect from ingested substance	164	68.62%	189	80.43%	116	78.91%	832	64.00%
M00-M99	Diseases of the musculoskeletal system	19	7.95%	16	6.81%	5	3.40%	94	7.20%
R00-R99	Abnormal clinical and laboratory symptoms	16	6.69%	10	4.26%	6	4.08%	81	6.20%
All others	All other ICD-10 with less than 1% for each of them	64	26.78%	20	8.51%	20	13.61%	294	22.60%
Total		239	100.00%	235	100.00%	147	100.00%	1301	100.00%

(1) Terminal tractor drivers, n = 239; (2) crane operators, n = 235; (3) stevedores, n = 147.

**Table 4:** Percentage of patients per shift.

Shift	I	II	III
Consultations	38%	34%	28%

**Table 5:** Distribution of ages.

Ages of Workers			
−29	30–49	50+	Total
286	803	211	1301

**Table 6:** Number of workers by gender.

Gender	Feminine	Masculine
Number of workers	157	1144

## Data Availability

Data are located in the MCT’s medical clinic. Specific requests can be sent to the corresponding author.
